# Post COVID-19 lung fibrosis and pleural effusion in geriatric patient

**DOI:** 10.11604/pamj.2021.40.32.30465

**Published:** 2021-09-10

**Authors:** Rashmi Ramesh Walke, Deepak Kumar Jain

**Affiliations:** 1 Department of Cardiovascular and Respiratory Physiotherapy, Ravi Nair Physiotherapy College, Wardha, Maharashtra, India,; 2 Department of Musculoskeletal Physiotherapy, Ravi Nair Physiotherapy College, Datta Meghe Institute of Medical Sciences, Wardha, Maharashtra, India

**Keywords:** SARS COVID, HRCT, CO-RAD, epidemiology, infectious disease, global health

## Image in medicine

An 81-year-old male who has hypertension in the last 15 years coming to our hospital with a chief complaint of severe breathlessness, chest pain with a history of fever spike in the last 20 days. When he underwent real-time reverse transcription-polymerase chain reaction (RT-PCR) test, the test comes positive with SARS COVID-19. Routine test high-resolution computed tomography (HRCT) showed approximately 75% of the left side and 50% of right lung involvement with CO-RAD 6 and HRCT severity score 20. HRCT showed multiple ill-defined patchy ground-glass opacity with consolidation and septal thickening in the bilateral lung field. There is a finding of fibrotic changes with tractional bronchiectasis in bilateral lung field with minimal pleural effusion.

**Figure 1:**
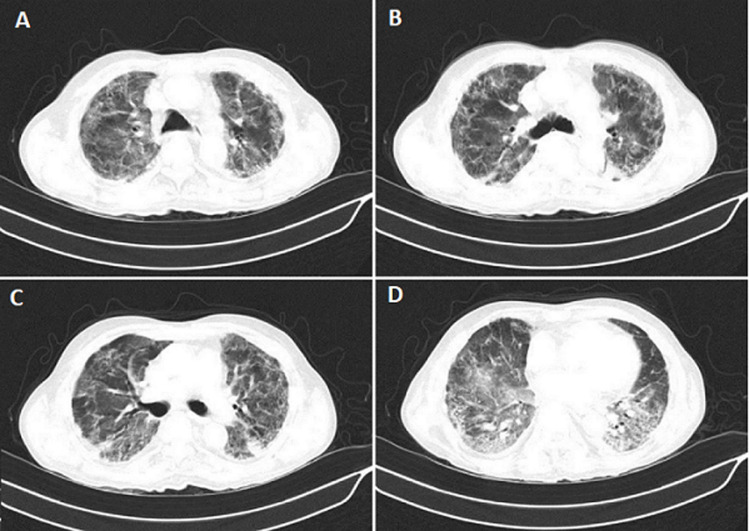
A) HRCT report showing fibrosis over the right lung; B) fibrosis over the left lung; C) bilateral consolidation; D) patchy ground glass opacity

